# Effects of apolipoprotein H downregulation on lipid metabolism, fatty liver disease, and gut microbiota dysbiosis

**DOI:** 10.1016/j.jlr.2023.100483

**Published:** 2023-12-14

**Authors:** Yaming Liu, Yiqun Zhao, Qiusong Liu, Binbin Li, P. Vineeth Daniel, Binbin Chen, Zeyi Wu

**Affiliations:** 1 Department of Gastroenterology and Hepatology, Xiamen University Zhongshan Hospital, Xiamen, FJ, China; 2 Department of Digestive Diseases, School of Medicine, Xiamen University, Xiamen, FJ, China; 3 Department of Tumor and Vascular Interventional Radiology, Xiamen University Zhongshan Hospital, Xiamen, FJ, China; 4 Division of Gastroenterology and Hepatology, Mayo Clinic, Rochester, MN, USA; 5 Department of Computer Science and Engineering, University of California, San Diego, CA, USA

**Keywords:** apolipoprotein H, cholesterol/metabolism, bile acids and salts, triglycerides, de novo lipogenesis, hepatocyte, gut microbiota

## Abstract

Apolipoprotein H (APOH) downregulation can cause hepatic steatosis and gut microbiota dysbiosis. However, the mechanism by which APOH-regulated lipid metabolism contributes to metabolic dysfunction–associated steatotic liver disease (MASLD) remains undetermined. Herein, we aim to explore the regulatory effect of APOH, mediated through various pathways, on metabolic homeostasis and MASLD pathogenesis. We analyzed serum marker levels, liver histopathology, and cholesterol metabolism–related gene expression in global *ApoH*^−/−^ C57BL/6 male mice. We used RNA sequencing and metabolomic techniques to investigate the association between liver metabolism and bacterial composition. Fifty-two differentially expressed genes were identified between *ApoH*^−/−^ and WT mice. The mRNA levels of de novo lipogenesis genes were highly upregulated in *ApoH*^−/−^ mice than in WT mice. Fatty acid, glycerophospholipid, sterol lipid, and triglyceride levels were elevated, while hyodeoxycholic acid levels were significantly reduced in the liver tissues of *ApoH*^−/−^ mice than in those of WT mice. Microbial beta diversity was lower in *ApoH*^−/−^ mice than in WT mice, and gut microbiota metabolic functions were activated in *ApoH*^−/−^ mice. Moreover, *ApoH* transcripts were downregulated in patients with MASLD, and APOH-related differential genes were enriched in lipid metabolism. Open-source transcript-level data from human metabolic dysfunction–associated steatohepatitis livers reinforced a significant association between metabolic dysfunction–associated steatohepatitis and APOH downregulation. In conclusion, our studies demonstrated that APOH downregulation aggravates fatty liver and induces gut microbiota dysbiosis by dysregulating bile acids. Our findings offer a novel perspective on APOH-mediated lipid metabolic dysbiosis and provide a valuable framework for deciphering the role of APOH in fatty liver disease.

## Graphical abstract

**Figure:**
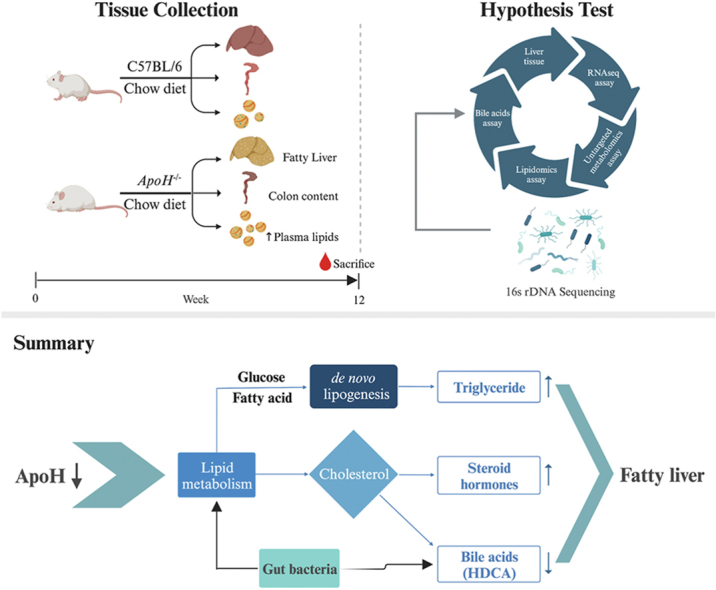

The prevalence of nonalcoholic fatty liver disease, recently renamed metabolic dysfunction-associated steatotic liver disease (MASLD), has increased globally over the last few decades (1, 2, 3). The number of patients with metabolic dysfunction–associated steatohepatitis (MASH) is rising, and consequently, cases of advanced liver disease are being reported with increasing frequency. Some studies have reported that steatohepatitis is an independent predictor of fibrosis (4). To combat the worsening burden of MASLD and related adverse outcomes, research elucidating its complex and disparate pathogenesis and identifying new therapeutic regimens is necessary. Notably, the gut microbiota plays a pivotal role in the pathophysiology of metabolic diseases, including MASLD, through the gut-liver axis (5, 6, 7). However, further research is needed to elucidate the mechanism underlying aberrant lipid metabolism and gut microbiota dysbiosis-mediated MASLD.

MASLD is characterized by the pathological accumulation of triglycerides (TGs) and other lipids in the liver (8, 9), and apolipoproteins play an essential role in lipoprotein metabolism (10). Apolipoprotein H (APOH) binds lipoproteins and activates lipoprotein lipases during TG metabolism. Several APOH isoforms are associated with apolipoprotein A, apolipoprotein B, HDL cholesterol, TG, and total cholesterol (TC) levels (11, 12, 13, 14, 15, 16, 17, 18, 19). In our previous studies, we found that APOH downregulation causes hepatocyte steatosis and that alcohol-dependent APOH downregulation exacerbates fatty liver and gut microbiota dysbiosis in mice (20, 21). However, the role of APOH in MASLD development has not been thoroughly examined. Therefore, using recent advances in analytical techniques to understand the APOH-mediated regulation of metabolic homeostasis in the liver may provide new insights into the mechanisms underlying MASLD.

In this study, we aimed to elucidate the potential regulatory role of APOH in MASLD development and investigate the association between APOH levels, hepatocyte steatosis, and gut microbiota dysbiosis. We analyzed liver function, the liver transcriptome, lipid metabolism, pathological changes, and alterations in intestinal flora in a global body *ApoH-*knockout mouse model, as well as assessed publicly available data for validation of our observations. Our findings provide a novel perspective on APOH-mediated lipid metabolic dysbiosis based on changes in gut microbiota, lipid metabolism, and bile acids.

## Materials and methods

### Animal care and generation of *ApoH*^−/−^ mice

All animal experiments were approved by the Institutional Animal Care and Use Committee of Xiamen University (XMULAC20210119) and were conducted according to the institutionally approved protocols and guidelines for animal care and use. The protocol for generating the *ApoH*^−/−^ mouse model was followed as reported in our previous studies (20, 21). Wild-type (WT; control) and *ApoH*^*−/−*^ C57BL/6 male mice were reared at the Xiamen University Laboratory Animal Center and euthanized at the age of 10–12 weeks (7–10 mice in each group, 4–5 mice per cage). The mice were fed routinely with a chow diet (Beijing KEAOXIELI Feed Co. Ltd., China) ad libitum. Mice were maintained in a regular 12 light/12 dark cycle animal facility. Before euthanasia, the mice were fasted for approximately 4 h. Mouse body and liver weights were determined (Table 1), and the serum, liver tissue, and colon contents were collected and stored at −80°C for subsequent analysis.

**Table 1:** Body and tissue mass in wildtype and *ApoH*^−/−^ mice

Parameters	Wild Type	*ApoH*^−/−^	*P*
Body mass (g)	24.84 ± 1.82	25.43 ± 0.91	0.299
Liver mass (g)	1.08 ± 0.09	1.19 ± 0.07	0.221
Liver mass/Body mass (%)	4.37 ± 0.31	4.67 ± 0.14	0.047∗

Data are means ± SD (n = 7 mice).

Wildtype and *ApoH*^−/−^ mice on a normal diet were examined at 3 months of age. ∗*P* < 0.05.

### Determination of serum alanine transaminase, aspartate transaminase, and hypersensitive C-reactive protein levels

Mouse peripheral blood (500 μl) was collected from the eyeballs and centrifuged at 3,000 rpm for 15 min at room temperature (approximately 23–25°C). The serum samples were then stored at −80°C until further use. Serum alanine transaminase (H-0041-20-52270), aspartate transaminase (H-0041-20-52274), and hypersensitive C-reactive protein (H-0043-20-81305) levels were quantified using a BS-240VET fully automatic biochemical analyzer (Mindray Global, Shenzhen, China) with a commercial assay kit (Mindray Global).

### Histopathological analysis

To determine the macroscopic changes in the tissue structures, 4% paraformaldehyde-fixed, paraffin-embedded mouse liver tissues were subjected to hematoxylin and eosin, periodic-acid Schiff, and Sirius Red staining. Additionally, the liver sections were incubated with antibodies against CD80 (Cat# ab254579, Abcam, Cambridge, UK), CD206 (Cat# 18704-1-AP, Proteintech, Rosemont, IL), and F4/80 (Cat# 29414-1-AP, Proteintech) at the manufacturer-recommended concentrations. CD80 and CD206 were used as M1 and M2 macrophage markers, respectively, whereas F4/80 was used as a pan-macrophage marker. The slides were scanned using an automated digital scanner (Pannoramic MIDI; 3DHISTECH, Budapest, Hungary), and the images were analyzed using CaseViewer 2.3.

### Quantification of liver TG, TC, and free fatty acid levels

Liver sections were collected and flash-frozen in liquid nitrogen. Thereafter, the frozen liver tissues were weighed and homogenized in absolute ethanol. The TG, TC, and free fatty acid (FFA) contents were quantified using commercial testing kits (Cat# A110-1-1, Cat# A111-1-1, and Cat# A042-2-1, respectively; Nanjing Jiancheng Bioengineering Institute, Nanjing, China) according to the manufacturer’s instructions.

### RNA sequencing and analysis

Frozen liver tissues from mice were randomly selected (each group, n = 3) and sent to the Beijing Genomics Institute for transcriptome sequencing. Beijing Genomics Institute automatic analysis software was used to analyze the RNA sequencing (RNA-seq) data. For the Kyoto Encyclopedia of Genes and Genomes (KEGG) pathway analysis, a chart was created with the enrichment ratio, which was calculated using the “Term Candidate Gene Num/Term Gene Num” represented on the *x*-axis and the KEGG pathway represented on the *y*-axis. The Q-values were obtained from the false discovery rate correction of the obtained *P-*values. Q-values ≤ 0.05 represented significant enrichment. A heatmap was then constructed to visualize the differentially expressed apolipoproteins and lipid metabolism–related genes in cholesterol metabolism.

### Reverse transcription real-time quantitative PCR

Total RNA was isolated from the liver tissues of the respective treatment groups using TRIzol reagent (Invitrogen, Carlsbad, CA) according to the manufacturer’s instructions. Thereafter, 1 μg of the total RNA was reverse transcribed to obtain the cDNA. The detailed reverse transcription real-time quantitative PCR (RT-qPCR) procedures were performed as outlined in a previous study (20). The primers used in this study are listed in Table 2.

**Table 2:** Mouse primers used in RT-qPCR analysis

Gene	Primers (5′- 3′)	
	Forward	Reverse
β-actin	AGGCCCAGAGCAAGAGAGGTA	GGGGTGTTGAAGGTCTCAAACA
APOH	TGCCATGAGACATACAAGCTG	GATCTTCACCCTCATCCCTTG
ACC	AAGGCTATGTGAAGGATGTGG	CTGTCTGAAGAGGTTAGGGAAG
CD36	GCGACATGATTAATGGCACAG	GATCCGAACACAGCGTAGATAG
FAS	CCCCTCTGTTAATTGGCTCC	TTGTGGAAGTGCAGGTTAGG
G6PC	TCTTGTGGTTGGGATTCTGG	CGGATGTGGCTGAAAGTTTC
MCAD	TGTTAATCGGTGAAGGAGCAG	CTATCCAGGGCATACTTCGTG
MTP	TCCACATACAGCCTTGACATC	TTAAGCCTTCCAGCCCTTG
PEPCK	CCATCCCAACTCGAGATTCTG	CTGAGGGCTTCATAGACAAGG
PPARα	CATTTCCCTGTTTGTGGCTG	ATCTGGATGGTTGCTCTGC
PPARγ	TGTTATGGGTGAAACTCTGGG	AGAGCTGATTCCGAAGTTGG
SCD	CTGACCTGAAAGCCGAGAAG	AGAAGGTGCTAACGAACAGG
SREBP1	CCATCGACTACATCCGCTTC	GCCCTCCATAGACACATCTG
SREBP1C	CCATCGACTACATCCGCTTC	GCCCTCCATAGACACATCTG
CYP7A1	AACGATACACTCTCCACCTTTG	CTGCTTTCATTGCTTCAGGG
CYP8B1	GTTTCTGGGTCCTCTTATTCCTG	TGGGAGTGAAAGTGAACGAC

### Metabolomic data analysis

Frozen liver tissues from the mice were randomly selected (each group, n = 6) and sent to Novogene Bioinformatics Technology Co., Ltd. (Tianjin, China) for metabolite extraction and ultra-high-performance liquid chromatography-mass spectrometry. An untargeted metabolomic assay was performed to explore the regulatory mechanism underlying the APOH downregulation-induced fatty liver disease. We further comprehensively analyzed the sterol lipid profiles in WT and *ApoH*^−/−^ mice. Targeted lipidomics and bile acid detection assays were also performed, and the NovoMagic automatic analysis software was used to analyze all the results (22). We assayed and analyzed the levels of primary and secondary bile acids. All 33 bile acid standards and six stable isotope-labeled standards were obtained from ZZ Standards Co., Ltd. (Shanghai, China). Analytical-grade ammonium acetate was obtained from Sigma–Aldrich (St. Louis, MO). Methanol (Optima LC-MS), acetonitrile (Optima LC-MS), and formic acid (Optima LC-MS) were purchased from Thermo Fisher Scientific (Waltham, MA). We used principal component analysis (PCA) to identify the similarities and differences between metabolite profiles, lipidomic profiles, and bile acid composition. Metabolites were annotated using the KEGG, Human Metabolome, and LIPID MAPS databases. A *t* test was used to calculate the statistical significance (*P*-value). Metabolites with a variable importance projection score >1, *P*-value <0.05, and fold change <0.833 and >1.2 were considered differential metabolites.

### Quantitative analysis of the 16S rRNA genes

Stool samples were collected from all mice and stored at −80°C until analysis. The samples (each group, n = 6) were sent to Novogene Bioinformatics Technology Co. Ltd. for microbial diversity detection. The V3–V4 region of the 16S rRNA gene was amplified as a bacterium-specific fragment. Amplicons were sequenced using the NovaSeq PE250 platform and analyzed using NovoMagic automatic analysis software. PCA was conducted to compare the beta diversity of the WT and *ApoH*^*−/−*^ groups at the phylum level. The diversity of the microbial community was measured using the Shannon–Weiner biodiversity index (Shannon index). The potential functions of the microbial communities from the sequencing data were predicted using Tax4Fun, followed by KEGG pathway analysis.

### Expression of *APOH* in patients with MASLD from the Gene Expression Omnibus database

All research involving human data was conducted according to the Declarations of Helsinki and Istanbul. The transcript-level expression of *APOH* in patients with MAFLD and MASH was analyzed using data from the open-source Gene Expression Omnibus (GEO) database (datasets GSE167523 and GSE162694). The *APOH*-associated enriched pathways were determined using KEGG analysis, and the upregulated and downregulated pathways were subsequently analyzed using gene set enrichment analysis. Additionally, correlations between liver fibrosis and inflammation scores were analyzed.

### Statistical analyses

Experimental results are expressed as the mean ± SEM from at least three independent experiments. A two-tailed Student’s *t* test was used, as appropriate, to test for significant differences between the WT and *ApoH*^*−/−*^ groups. A one-way ANOVA and nonparametric test were performed owing to the skewed distribution of human sample data. Correlations between two indicators were tested using Spearman’s rank correlation. Statistical significance was set at *P* < 0.05.

## Results

### *ApoH*^−/−^ mice demonstrate fatty liver phenotype

**Fig. 1:**
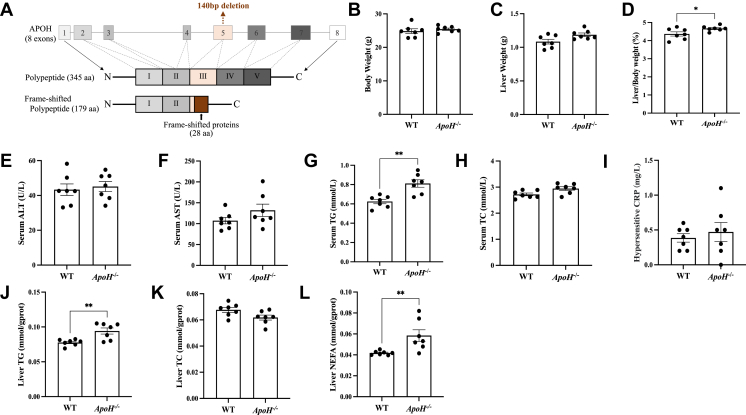
*ApoH*-knockout mice exhibit signs of fatty liver. A: C57BL/6J mice (n = 7) were used to generate an *ApoH-*knockout mouse model using CRISPR, wherein 140 bp were deleted from the transcriptional X1 exon 3. Twelve-week-old mice were euthanized to determine the (B) body and (C) liver weight, (D) the liver-to-body weight ratio, and serum levels of (E) alanine transaminase (ALT), (F) aspartate transaminase (AST), (G) total triglyceride (TG), (H) total cholesterol (TC), and (I) C-reactive protein (CRP). J–L: Liver triglycerides, total cholesterol, and nonesterified fatty acid levels in WT and *ApoH*^−/−^ mice were normalized to the liver protein content. Data are presented as the mean ± SEM. ∗*P* < 0.05. NEFA, nonesterified fatty acids. ∗*P* < 0.05, ∗∗*P* < 0.01.

Fig. 1A shows a schematic of *ApoH* knockout. No significant difference was observed in the body or liver weights between *ApoH*^−/−^ and WT mice. However, the liver-to-body mass ratio was greater in *ApoH*^−/−^ mice than in WT mice (*P* < 0.05) (Fig. 1B–D; Table 1). Serum TG levels and liver TG and FFA contents were significantly higher in *ApoH*^−/−^ mice than in WT mice (*P* < 0.05) (Fig. 1G, J, L). No significant differences were observed in serum alanine transaminase, aspartate transaminase, TC, and C-reactive protein levels or liver TC levels between *ApoH*^−/−^ and WT mice (*P* > 0.05; Fig. 1E, F, H, I, K).

**Fig. 2:**
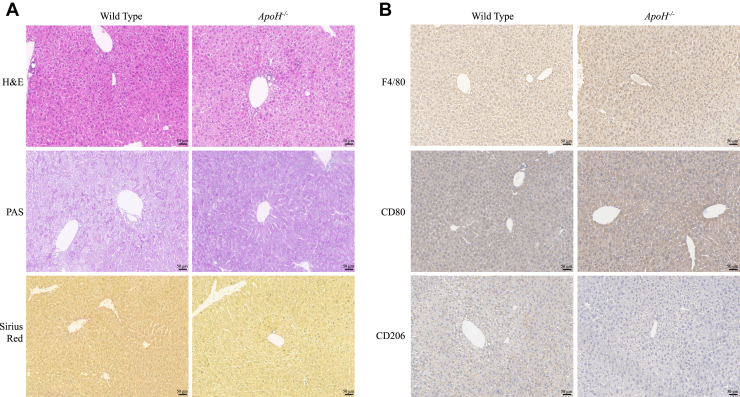
*ApoH*-knockout mouse liver tissue staining. A: Hematoxylin and eosin, periodic-acid-Schiff, and Sirius Red staining of liver tissues. B: Polarized macrophages were examined using immunohistochemical staining with antibodies against F4/80, CD206, and CD80. Images show 20× magnification.

Additionally, we observed apparent steatosis in the hematoxylin and eosin–stained liver sections of *ApoH*^−/−^ mice (Fig. 2A). No significant difference was observed in macrophage polarization between *ApoH*^−/−^ and WT mice, as indicated by the macrophage markers F4/80, CD206, and CD80 (Fig. 2B). We also did not find any differences in glycogen levels, fibrosis, or inflammation between the liver tissues of *ApoH*^−/−^ and WT mice. Therefore, based on these findings, we infer *ApoH*^−/−^ mice demonstrate fatty liver phenotype.

### *ApoH*^−/−^ mice have dysregulated hepatic lipid metabolism

**Fig. 3:**
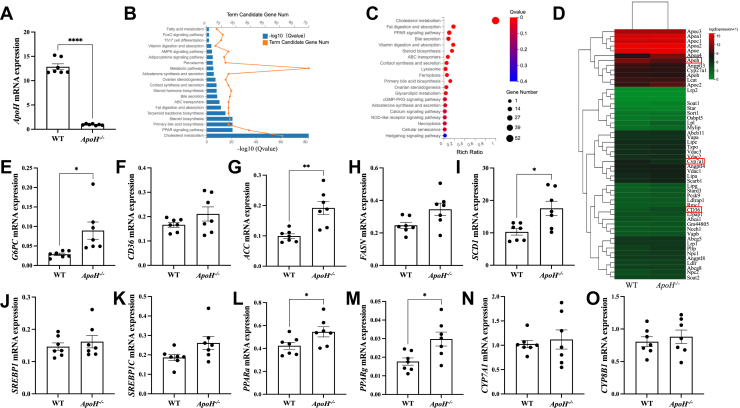
RNA-seq and pathway analysis of the liver sections from *ApoH*^−/−^ mice. A: *ApoH* mRNA levels were assayed using RT-qPCR. B: Cholesterol-related pathways were analyzed based on the Kyoto Encyclopedia of Genes and Genomes (KEGG) pathway database. C: Bubble diagram representing the cholesterol metabolism pathways in *ApoH*^−/−^ mice compared with those of the WT mice. D: Heatmap representing the 52 differentially expressed cholesterol metabolism genes between *ApoH*^−/−^ and WT mice. E–M: Relative expression of genes involved in regulating lipogenesis, including (E) *G6pc*, (F) *Cd36*, (G) *Acc*, (H) *FASNasn*, (I) *SCDcd1*, (J) *Srebp1*, (K) *Srebp1C*, (L) *Pparα*, and (M) *Pparγ.* N, O: Relative expression of (N) *Cyp7a1* and (O) *Cyp8b1*, which are the genes involved in regulating bile acid synthesis. Data are presented as the mean ± SEM. ∗*P* < 0.05, ∗∗*P* < 0.01, ∗∗∗∗*P* < 0.0001.

*ApoH* mRNA expression, as determined by RNA-seq of liver sections from WT and *ApoH*^−/−^ mice, is illustrated in Fig. 3A. KEGG analysis revealed the enrichment of cholesterol-related metabolic pathways in *ApoH*^−/−^ mice (Fig. 3B, C). RNA-seq data from mouse liver tissues indicated that *ApoH* was involved mainly in cholesterol metabolism pathways, including “fat digestion and absorption,” “PPAR signaling,” “bile secretion,” “steroid biosynthesis,” “primary bile acid biosynthesis,” and “glycerolipid metabolism” (Fig. 3C). According to the heatmap constructed to determine the differentially expressed apolipoproteins and lipid metabolism-related genes between WT and *ApoH*^−/−^ mice, apolipoprotein-related genes (*ApoA, ApoB, ApoC*, and *ApoE*), CYP450-related genes (*Cyp7a1* and *CYP27A1Cyp27a1*), and de novo lipogenes (*Cd36*) were identified (Fig. 3D). Subsequently, RT-qPCR analysis of de novo lipogenesis-related genes indicated that the mRNA levels of *G6pc*, *ACCcc*, *FASNasn*, *Scd1*, *PPARα*, and *PPARγ* were significantly upregulated in *ApoH*^−/−^ mice compared with those in WT controls (*P* < 0.05) (Fig. 3E–M). No significant differences were observed in the mRNA expression of *Cyp7a1* or *Cyp8b1* between the two groups (*P* > 0.05; Fig. 3N, O). These results show that *ApoH* deletion primarily affects liver lipid metabolism by modulating cholesterol metabolism, fatty acid metabolism, and de novo lipogenesis.

**Fig. 4:**
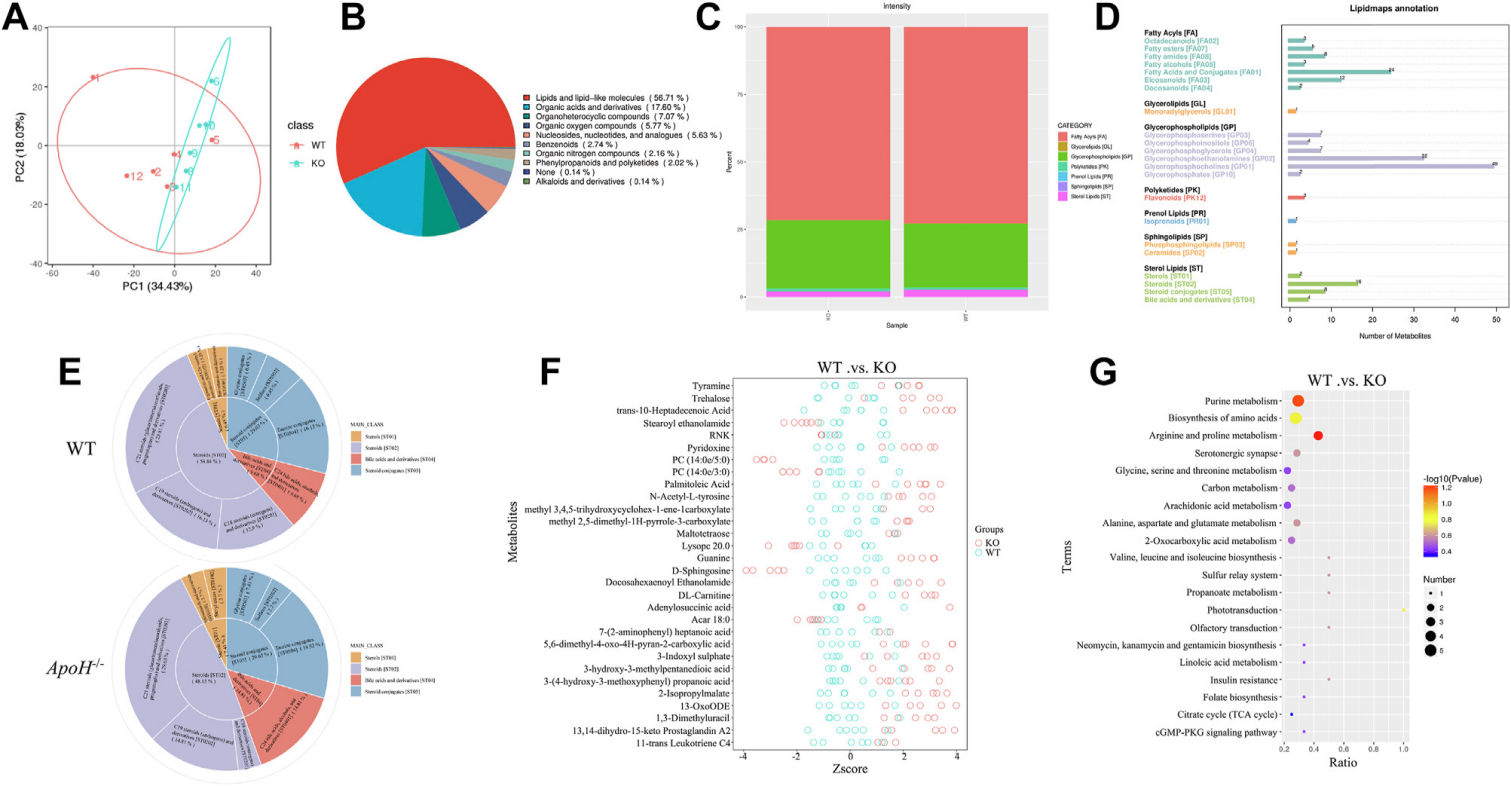
Untargeted metabolome analysis of the liver sections of *ApoH*^−/−^ mice. A: Principal component analysis (PCA) of the differential metabolites from the untargeted metabolomic assay. B: Pie chart representing the category variables of the metabolites in WT and *ApoH*^−/−^ mice. C: Intensity-stacked bar chart comparing different class variables of the WT and *ApoH*^−/−^ mice. D: LIPID MAPS annotations indicating the differential metabolites between WT and *ApoH*^−/−^ mice. E: Pie charts displaying the profiles of sterol lipids in WT and *ApoH*^−/−^ mice. F: Z-score diagram representing the differential metabolites between WT and *ApoH*^−/−^ mice. G: Differential metabolite enrichment using KEGG pathway analysis.

Next, the untargeted metabolomic assay and PCA showed that lipids and lipid-like molecules (56.71%) were the predominant metabolites in the liver tissues of *ApoH*^−/−^ mice, followed by organic acids and derivatives (17.60%), organoheterocyclic compounds (7.07%), and organic oxygen compounds (5.77%) (Fig. 4A, B). The components and proportions of lipid metabolites in WT and *ApoH*^−/−^ mice are shown in Fig. 4C. The LIPID MAPS annotation showed that fatty acids, glycerolipids, glycerophospholipids, and sterol lipids were the predominant lipid categories in *ApoH*^−/−^ mice (Fig. 4D). As cholesterol metabolism is a pivotal lipid metabolism regulatory pathway in the progression of hepatic steatosis, the profiles of sterol lipids in WT and *ApoH*^−/−^ mice were further analyzed. We found that the proportion of bile acids and derivatives was notably increased in *ApoH*^−/−^ mice than in WT mice (14.81% vs. 9.68%, respectively; Fig. 4E). Analysis of the differential metabolites between WT and *ApoH*^−/−^ mice (Fig. 4F) revealed the enrichment of purine, amino acid, and carbon metabolism pathways (Fig. 4G). Therefore, APOH deficiency impaired liver lipid metabolism in mice.

### Impaired hepatic lipid metabolism in *ApoH*^−/−^ mice accounts for altered cholesterol and bile acid metabolism

**Fig. 5:**
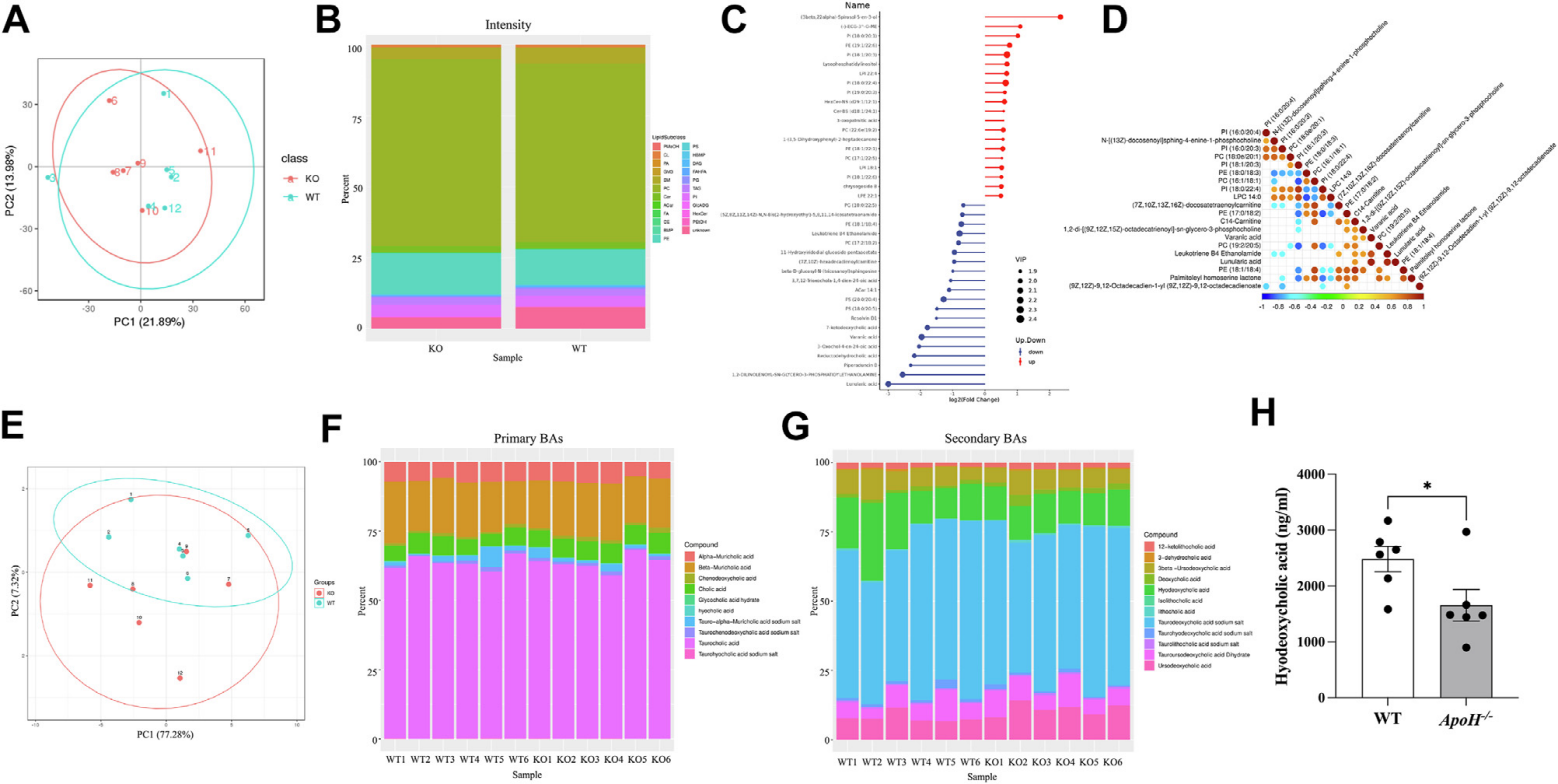
Impaired hepatic lipid metabolism in *ApoH*^−/−^ mice accounts for altered cholesterol and bile acid metabolism. A: Differential metabolites were analyzed via a lipidomic assay using principal component analysis (PCA). B: Intensity-stacked bar chart comparing the category variables based on a lipid classification system between WT and *ApoH*^−/−^ mice. C: Stem diagram representing the differential metabolites between WT and *ApoH*^−/−^ mice. D: Class-wise correlation of all differential lipid metabolites between WT and *ApoH*^−/−^ mice. E: Differential bile acids were analyzed using PCA. F and G: Intensity-stacked bar chart comparing the profiles and levels of primary bile acids (BAs) and secondary BAs in WT and *ApoH*^−/−^ mice. H: Hyodeoxycholic acid was identified as a differential BA between WT and *ApoH*^−/−^ mice. ∗*P* < 0.05.

Targeted lipidomics and bile acid analyses were performed to elucidate the mechanism underlying APOH-mediated lipid metabolism. The overall similarities and differences between the samples determined through PCA are shown in Fig. 5A, E. The lipid components in WT and *ApoH*^−/−^ mice were analyzed, and the proportion of total phosphatidylserine was significantly increased in *ApoH*^−/−^ mice (Fig. 5B). The detailed differential lipid metabolites are shown in Fig. 5C. Phosphatidylcholine (PC), phosphatidylethanolamine (PE), phosphatidylinositol, and sphingomyelin (SM) levels were markedly higher, whereas phosphatidylserine, 7-ketodeoxycholic acid, and reductodehydrocholic acid levels were lower in *ApoH*^−/−^ mice than in WT mice. Then, correlation analysis was performed among these metabolites. We found that PC was positively correlated with phosphatidylinositol and PE (Fig. 5D). Next, we analyzed the components of primary and secondary bile acids in WT and *ApoH*^−/−^ mice (Fig. 5F, G) and found that the level of hyodeoxycholic acid (HDCA) was significantly lower in *ApoH*^−/−^ mice than in WT mice (*P* < 0.05; Fig. 5H). Taken together, metabolomics tests further clarified that APOH deficiency impaired liver lipid metabolism, especially activated cholesterol metabolism via dysregulated bile acid metabolism.

### Altered gut microbiome correlates *ApoH* deficiency with dysregulated bile acid metabolism

**Fig. 6:**
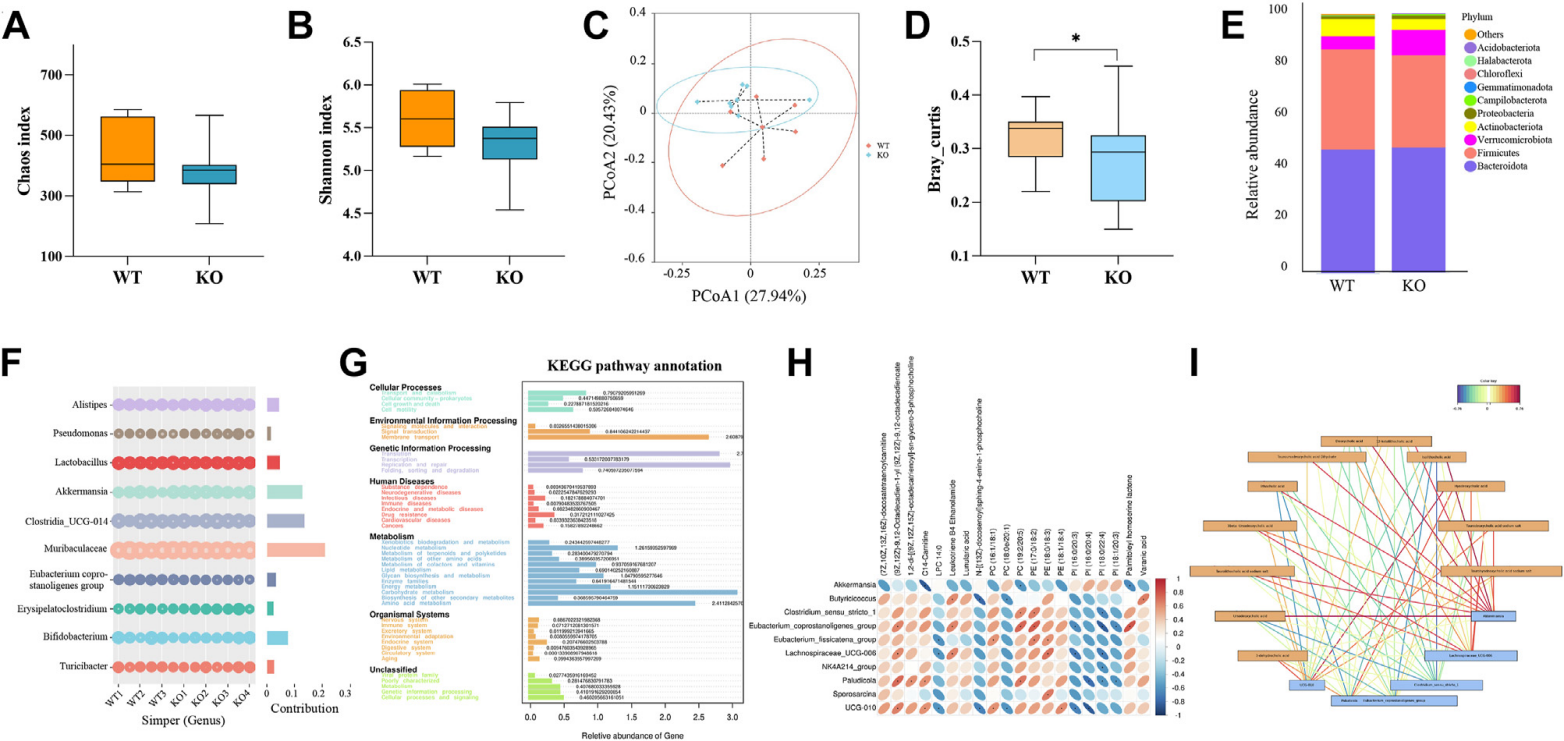
Altered gut microbiome correlates *ApoH* deficiency with dysregulated bile acid metabolism. Measurements of the alpha diversity in WT and *ApoH*^−/−^ mice, comprising the (A) Chao1 index and (B) Shannon-Weiner biodiversity index (Shannon index). *P* > 0.05. C: Principal coordinate analysis (PCA) of the beta diversity comparison using Bray-Curtis distances, revealing the separation of microbial communities based on the *ApoH-*knockout genotype (*P* > 0.05). D: Beta diversity in WT and *ApoH*^−/−^ mice (*P* < 0.05). Bacterial composition at the phylum (E) and genus (F) levels in WT and *ApoH*^−/−^ mice. G: Predictive functional profiling of the microbial communities using Tax4Fun and KEGG pathway enrichment analyses. H: Correlation between gut bacteria and untargeted liver metabolomic changes. I: Correlation between gut bacterial and liver bile acid profiles and levels. ∗*P* < 0.05.

With an aim to understand the source of increasing HDCA, we assessed the gut microbiome via 16S rRNA sequencing. The sequencing data indicated no significant difference in alpha diversity between the *ApoH*^−/−^ and WT mice (*P* > 0.05; Fig. 6A, B). However, PCA revealed a significant difference in beta diversity between groups (*P* < 0.05; Fig. 6C, D). Total bacterial composition and abundance at the phylum and genus levels were lower in *ApoH*^−/−^ mice than in WT mice (Fig. 6E, F). At the phylum level, *ApoH*^−/−^ mice showed a markedly higher abundance of Bacteroidetes, Verrucomicrobiota, and Proteobacteria and a lower abundance of Firmicutes and Actinobacteria than WT mice. At the genus level, *ApoH*^−/−^ mice showed a higher abundance of *Clostridia*, *Akkermansia*, *Alistipes*, *Erysipelatoclostridium*, and *Turicibacter* and a lower abundance of *Muribaculacease*, *Bifidobacterium*, *Lactobacillus*, and *Eubacterium* than WT mice. Tax4Fun and KEGG analyses of the differential bacteria between *ApoH*^−/−^ and WT mice revealed that the gut microbiota mainly regulated metabolic functions. Further analysis using untargeted metabolomic assay data indicated a correlation between bacteria and differential metabolites (Fig. 6G, H). We also found that HDCA levels were positively correlated with the abundance of *Akkermansia* and negatively correlated with that of *Clostridium* (Fig. 6I). Notably, APOH deficiency caused gut microbiota dysbiosis in mice which was closely correlated with bile acid HDCA levels.

### *APOH* expression in hepatic steatosis patient data from the GEO database

**Fig. 7:**
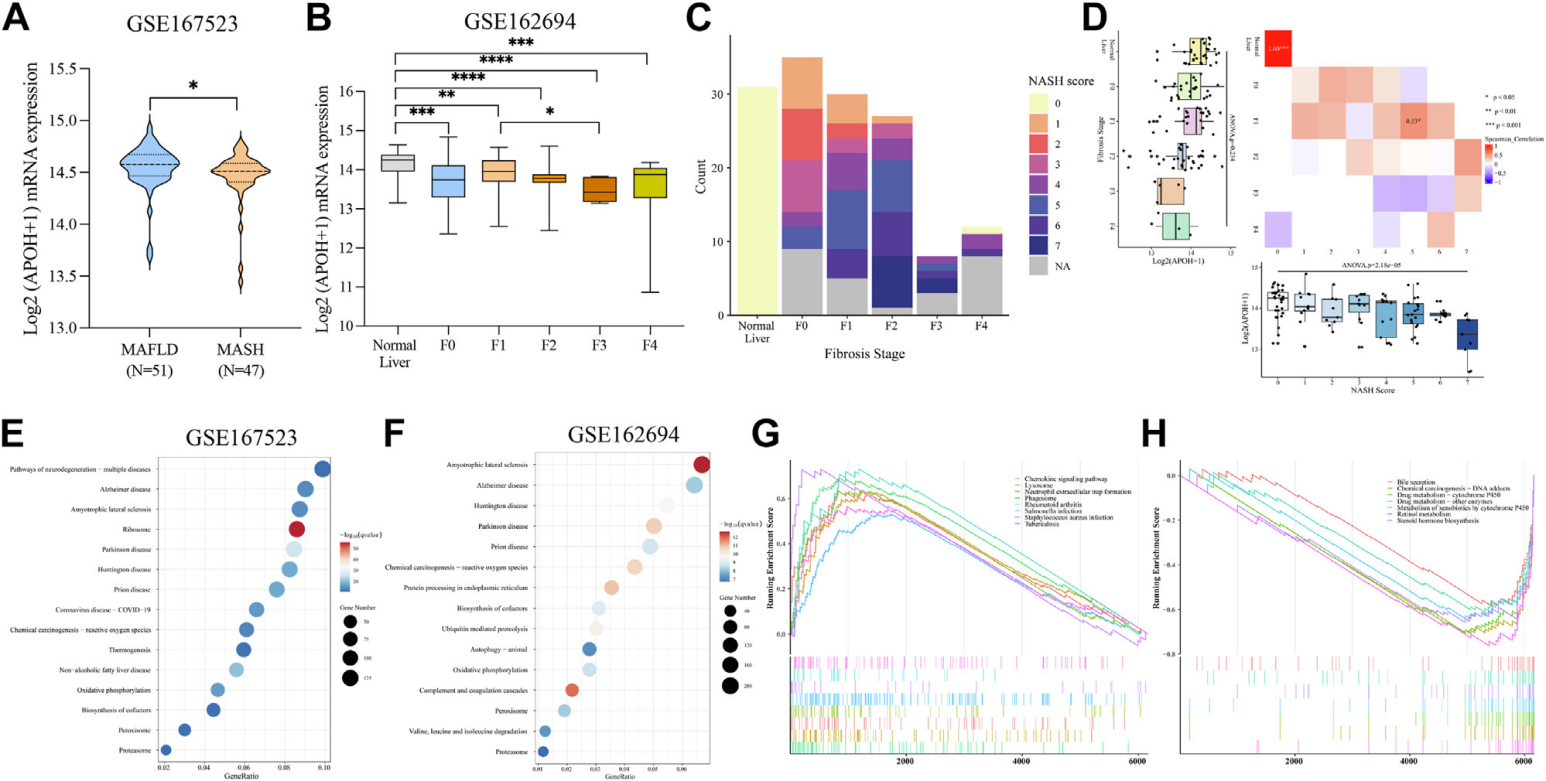
*APOH* expression in hepatic steatosis patient data from the GEO database. A and B: *APOH* expression data at the transcriptional level in patient liver tissues obtained from two GEO datasets: (A) GSE167523 and (B) GSE162694. C and D: Number and percentage of patients with different metabolic dysfunction associated steatohepatitis (MASH) scores during the full spectrum of liver fibrosis among patients with MASLD whose data were hosted on the Gene Expression Omnibus database (GSE162694 dataset). E and F: KEGG pathway enrichment of *APOH*-related genes obtained from two GEO datasets: E: GSE167523 and (F) GSE162694. G and H: Upregulated (G) and downregulated (H) *APOH*-related gene enrichment pathways identified using gene set enrichment analysis. ∗*P* < 0.05, ∗∗*P* < 0.01, ∗∗∗*P* < 0.001, ∗∗∗∗*P* < 0.0001.

We next considered to interrogate publicly available human MASH patients liver transcriptome and correlate a potential association with our APOH observations. In the GEO dataset GSE167523, *APOH* expression in the livers of patients with MASH was lower than in the livers of patients with MASLD (*P* < 0.05; Fig. 7A). In the GSE162694 dataset, *APOH* expression gradually decreased in patients with hepatic steatosis during the progression of liver fibrosis (*P* < 0.05; Fig. 7B). Further analysis indicated that liver inflammation increased with the pathological stage of fibrosis in patients with MASLD (Fig. 7C). In addition, inflammation and fibrosis synergistically affected APOH expression (Fig. 7D). Next, KEGG analysis showed that *APOH*-associated genes were enriched in the “nonalcoholic fatty liver disease” pathway for the GSE167523 dataset (Fig. 7E), whereas genes from the GSE162694 dataset were mainly enriched in “protein processing in endoplasmic reticulum,” “oxidative phosphorylation,” “peroxisome,” and “proteasome” pathways (Fig. 7F). Furthermore, gene set enrichment analysis showed that the downregulated genes in patients with fatty liver disease were mainly enriched in the following pathways: “bile secretion,” “chemical carcinogenesis-DNA adducts,” “drug metabolism-CYP450 and other enzymes,” “metabolism of xenobiotics by CYP450,” “retinol metabolism,” and “steroid hormone biosynthesis” (Fig. 7G, H). Taken together, we observe that APOH expression was downregulated in patients with MASLD which was synergistically affected by liver pathologic inflammation and fibrosis.

## Discussion

In this study, we mapped the transcriptional and metabolic profiles of *ApoH* downregulation-induced fatty liver in mice to elucidate the underlying mechanisms by which APOH regulates lipid metabolism and affects the pathogenesis of MASLD. The graphical abstract summarizes the experimental procedures and key findings.

In our mouse model, we found that *ApoH*^*−/−*^ mice clearly displayed fatty liver disease and increased serum TG levels than WT mice. In our previous study, we constructed chronic-plus-binge mouse model and HBV replication mouse model on the background of the *ApoH*^*−/−*^ mice. However, this observation was slightly different from the previously reported results on spontaneous steatohepatitis in *ApoH*^*−/−*^ mice (20, 21). This discrepancy could be attributed to the fact that the *ApoH*^*−/−*^ mice used in this study were euthanized at approximately 12 weeks of age, whereas in previous studies, *ApoH*^*−/−*^ mice were euthanized at an older age when the disease models were established. In the present study, we attempt to clarify a deep underlying linkage mechanism of *ApoH* gene knockout–induced metabolic dysbiosis from gut-liver axis.

At first, considering the spectrum of MASLD, including fatty liver, steatohepatitis, fibrosis, cirrhosis, or liver cancer, we speculate that APOH downregulation-induced fatty liver might be an early pathologic change and could evolve into hepatitis or fibrosis during disease progression. In this study, we explored the potential mechanism by which *ApoH* deletion induced fatty liver disease. During lipid metabolism, APOH binds to several lipoproteins and activates lipoprotein lipase, which is associated with TG metabolism and cholesterol transport (17). Validation of the RNA-seq analyses using RT-qPCR indicated that *ApoH* downregulation activated de novo lipogenesis and induced abnormal cholesterol metabolism. The results are also consistent with our previous in vitro experiment (21, 23). Thus, we deduced that the above results were the initiating factors causing fatty liver disease in *ApoH*^*−/−*^ mice.

Next, we investigated the effect of APOH downregulation on hepatic lipid metabolism. Untargeted metabolomic analysis indicated that diminished APOH levels facilitated FFA and glycerophospholipid metabolism, particularly altering steroid, and bile acid metabolism. Lipidomic data revealed notable differences in phospholipid levels between *ApoH*^*−/−*^ and WT mice. Notably, phospholipid metabolism regulates lipid, lipoprotein, and whole-body energy metabolism. For example, PC is synthesized in the liver via the choline pathway or the methylation of PE via phosphatidylethanolamine N-methyltransferase. An abnormal PC-to-PE ratio is associated with hepatocyte steatosis (24). SM effectively absorbs choline-containing compounds from phospholipid digestion in the intestinal tract; these compounds are subsequently used for PC synthesis. Alterations in SM levels are associated with nonalcoholic steatohepatitis onset and progression (25). Our findings indicated that APOH downregulation induces or exacerbates fatty liver by altering phospholipid metabolism. Furthermore, targeted bile acid detection revealed that HDCA levels were significantly decreased in the liver tissue of *ApoH*^*−/−*^ mice compared with those in the liver tissue of WT mice. HDCA, a secondary bile acid, is a metabolic byproduct of intestinal bacteria and activates liver X receptors and G protein-coupled receptors, such as Takeda G-protein receptor 5, to regulate lipid metabolism in the liver (26). Furthermore, HDCA is a weak agonist of liver X receptor α and Takeda G-protein receptor 5. HDCA exerts hypolipidemic effects in mice by reducing the expression of sterol regulatory element-binding protein-1c, acetyl-coenzyme A synthase, FAS, and stearoyl-CoA desaturase in the liver (26). In light of this, our findings suggest that APOH downregulation reduces HDCA production to promote hepatic steatosis by regulating the gut-liver axis.

Because gut-liver axis regulation and gut microbiota dysbiosis have been highlighted as crucial pathogenic factors in MASLD (26–28), we assessed the diversity of the gut bacterial community and differences in bacterial species abundance between *ApoH*^−/−^ and WT mice. The total bacterial diversity and abundance at the phylum and genus levels were significantly lower in *ApoH*^−/−^ mice than those in WT mice, and the microbial communities were mainly associated with metabolic functions, consistent with the previously reported findings (27). We speculate that *ApoH* gene knockout causes aberrant lipid metabolism in the liver and subsequently disturbs the gut microbiota homeostasis, which further influences metabolic dysregulation in the intestine and modulates liver metabolism through the feedback of enterohepatic circulation. In future research, we intend to utilize germ-free mice based on *ApoH*^−/−^ mice to further explore the regulatory mechanism of enterohepatic circulation. In addition, we intend to supplement exogenous APOH in mice with fatty liver disease and observe the subsequent changes in liver injury.

In conclusion, in the present study, we aimed to elucidate the precise regulatory mechanism of APOH in hepatic steatosis. Overall, our results suggest that a reduction in APOH levels causes TG aggregation in the liver, disordered cholesterol-derived bile acid metabolism, and microbial dysbiosis, leading to gut-liver axis dysregulation. Through these alterations, APOH downregulation aggravates fatty liver and is involved in MASLD pathogenesis. Our findings provide a novel perspective on APOH-mediated lipid metabolic dysbiosis based on changes in the gut microbiota, lipid metabolism, and bile acids metabolism. Despite the importance of these findings, this study had certain limitations. First, bile acid components of mice and humans is different; hence, the observations recorded for mice might not be very relevant in humans. Hence, we plan to enroll patient cohorts for further translational studies. Second, the study findings may not adequately reflect the findings observed in clinical settings; therefore, additional in-depth studies investigating the role of APOH using animal models of fatty liver disease are needed. Overall, this study provides a valuable framework for deciphering the role of APOH in the pathogenesis of fatty liver disease and enabling newer therapeutic explorations.

## Data availability

Liver section RNA-seq, liver metabolomics, and stool sample 16S rRNA sequencing were performed by Beijing Genomics Institute and Tianjin Novogene Bioinformatic Technology Co., Ltd. The datasets generated and analyzed herein are available in the NCBI SRA repository (https://submit.ncbi.nlm.nih.gov/subs/sra/). All data related to this study are available upon request to the corresponding author (Dr Yaming Liu; yaming0856@gmail.com).

## Supplemental data

This article contains supplemental data.

## Conflict of interest

The authors declare that they have no conflicts of interest with the contents of this article.
