# Supplementary figures and images for: Effects of apolipoprotein H downregulation on lipid metabolism, fatty liver disease, and gut microbiota dysbiosis
Source: J Lipid Res. 2023 Dec 14;65(1):100483. doi: 10.1016/j.jlr.2023.100483 (PMC10818206; doi:10.1016/j.jlr.2023.100483)

# Supplement Figure 1

A

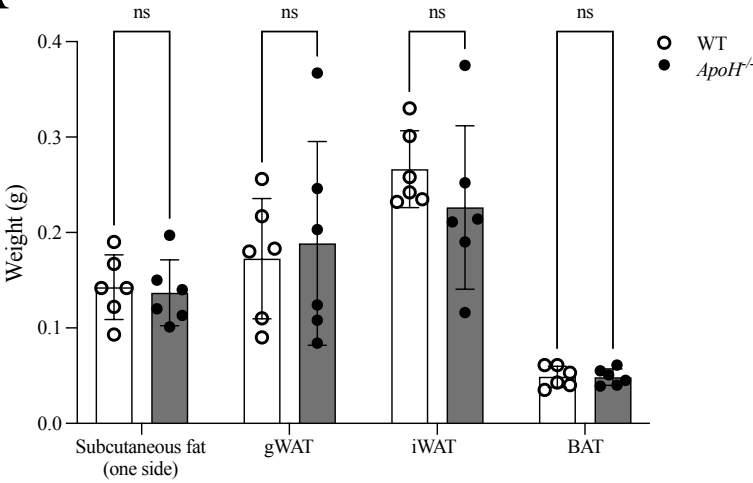

Supplement: Supplemental Figure S1 [file mmc1.pdf]
